# Supplementary material for: Linguistic analysis of health anxiety during the COVID-19 pandemic
Source: PLoS One. 2024 Feb 26;19(2):e0299462. doi: 10.1371/journal.pone.0299462 (PMC10896548; doi:10.1371/journal.pone.0299462)
Supplement: Attachment — Submitted filename: Reviewer_Response.docx [file pone.0299462.s002.docx]

**Manuscript Number:** PONE-D-23-27003

**Title:** Linguistic analysis of health anxiety during the COVID-19 pandemic

Reviewer #1:

1. The use of ''health anxiety'' is still somehow confusing and most professionals trained in mental health might still not be able to understand it. Professionals in mental health and psychiatry communicate either by using Diagnostic and statistical manual (DSM) or the international classification of diseases (ICD). According to the authors definitions of heath anxiety, it might qualify as hypochondriasis or illness anxiety disorder (ICD and DSM 5 respectively). The authors should consider adopting the DSM/ICD nomenclature or adding text to explain the equivalent of health anxiety in ICD or DSM.

**RESPONSE: We have added clarification on the definition of health anxiety as a transdiasgnostic vulnerability factor exhibited in multiple DSM-5 diagnoses. The revised text reads (line 62) as follows:** *Clinically elevated health anxiety is regularly expressed as persistent worry and maladaptive beliefs about health-related stimuli, along with excessive engagement in subsequent compensatory behaviors (e.g., excessive handwashing, reassurance-seeking). Lifetime prevalence of clinically elevated health anxiety ranges from 1.3% to 10% in the general population [4]. Studies have shown that elevated health anxiety is a transdiasgnostic vulnerability factor exhibited in multiple DSM-5 psychiatric disorders such as illness anxiety disorder and somatic symptom disorder as well as panic disorder and obsessive-compulsive disorder [4, 5].*
